# Fine dust risk perception, perceived restorativeness, and environmental policy support among park visitors

**DOI:** 10.3389/fpsyg.2025.1600895

**Published:** 2025-10-13

**Authors:** Jee In Yoon, Jiwon Yi, Jinyoung Joo

**Affiliations:** ^1^Department of Coaching, College of Physical Education, Kyung Hee University, Yongin-si, Republic of Korea; ^2^Center for Happiness Studies, Seoul National University, Seoul, Republic of Korea

**Keywords:** fine dust risk perception, perceived restorative environment, environmental policy, outdoor recreation, Jeju

## Abstract

**Objectives:**

Fine dust (PM2.5) pollution has emerged as a severe environmental and public health issue in South Korea, affecting respiratory health and reducing outdoor physical activity levels. Given these concerns, individuals increasingly seek restorative natural environments to mitigate the psychological and physical effects of poor air quality. This study examines how fine dust risk perception influences individuals' experiences in perceived restorative environments (PRE) and their intention to support environmental policies. Specifically, this study enhances the understanding of how restorative experiences in urban parks and concerns about environmental issues influence recreationists' perspectives on environmental policies.

**Methods:**

This study was conducted at Jeju Gotjawal Provincial Park, a well-preserved forest ecosystem known for its ecological importance. A total of 408 visitors were surveyed using purposive sampling. Participants completed a structured questionnaire assessing their fine dust risk perception, perceived restorative experience in nature, and intention to support environmental policies. Data was analyzed using confirmatory factor analysis (CFA) and structural equation modeling (SEM) to test the proposed hypotheses. In addition, a bootstrap analysis was conducted to examine the mediating effects.

**Results:**

The findings indicate that individuals who perceive fine dust pollution as a serious issue are more likely to experience a heightened sense of psychological and physical restoration in natural settings. Furthermore, those who find natural environments more restorative tend to express stronger support for environmental policies, suggesting that their positive experiences in nature reinforce their commitment to sustainability. The study also found that perceived restorativeness plays a key role in connecting fine dust risk perception to environmental policy support, emphasizing the importance of personal experiences in shaping pro-environmental attitudes.

**Conclusions:**

These results highlight the role of nature-based experiences in fostering both psychological well-being and environmental engagement. As air pollution continues to pose health risks, providing access to clean, green spaces can be an effective intervention for improving public health and encouraging sustainability advocacy. Policymakers and urban planners should integrate green spaces into urban planning to enhance citizens' environmental awareness and engagement. Future studies should explore longitudinal effects and cross-regional comparisons to validate these findings further.

## 1 Introduction

Over the past two decades, fine dust, also known as particulate matter (PM), has emerged as a significant environmental and public health issue in South Korea (Yoo, 2019). Fine dust consists of liquid droplets and solid particles suspended in the atmosphere and is primarily caused by the combustion of fossil fuels and transboundary pollution (Vohra et al., 2021). Due to South Korea's geographical location and industrial activities, exposure to fine dust has been an unavoidable concern, severely impacting public health. Numerous studies have linked PM exposure to respiratory diseases, cardiovascular complications, and even premature mortality (Kim and Shin, 2021). According to recent research, an increase of 1 microgram per cubic meter in PM2.5 concentration leads to 31.2 additional deaths per million people annually in South Korea (Jeong and Ra, 2022). Especially infants and individuals with pre-existing health conditions being the most vulnerable (Jeong and Ra, 2022).

In response to deteriorating air quality, many South Koreans have sought cleaner and more refreshing environments to engage in physical activities during their leisure time. Previous studies suggest that environmental pollution, particularly air pollution, negatively affects outdoor physical activity levels, such as walking and exercising (Kim and Shin, 2021). Conversely, natural settings such as forests and parks provide an opportunity for individuals to escape polluted urban areas and restore their physical and psychological well-being (Marselle et al., 2013). Jeju Gotjawal Provincial Park, an ecologically significant and relatively unpolluted area, serves as a critical recreational space for both residents and tourists seeking respite from urban air pollution. The park's unique geological and ecological features, formed by lava flows, support diverse flora and fauna exclusive to Jeju Island.

While extensive literature has documented the psychological and physiological benefits of restorative natural environments (Frumkin et al., 2017; Hartig et al., 2001), limited empirical work has examined how individuals' environmental risk perceptions—such as concerns about fine dust—shape or enhance their restorative experiences in nature. The concept of perceived restorativeness refers to the process by which individuals recover from stress and mental fatigue while engaging with nature (Kaplan and Kaplan, 1989). This restorative experience improves psychological resilience and contributes to mental health and overall life satisfaction (Marselle et al., 2013). Additionally, nature exposure is linked to increased environmental awareness and pro-environmental attitudes. Research has demonstrated that individuals who frequently interact with natural environments tend to exhibit stronger environmental concerns and behaviors that support sustainability (Schuttler et al., 2018).

However, few studies have empirically linked environmental risk perception—such as air pollution anxiety—with restorative experiences and subsequent policy support within a single analytical framework. Existing studies have tended to isolate these constructs, thereby missing the complex psychological pathways that connect environmental threats to policy-relevant behavior. For example, Kim (2021) showed that higher levels of air pollution led to reduced outdoor mobility, particularly among vulnerable groups such as the elderly. Yoo (2021) also found that public access to real-time air quality data leads to significant behavioral changes, such as reduced outdoor activity. Chung et al. (2023) showed further demonstrated that eco-anxiety is key predictor of environmental sustainability interest, highlighting the psychological salience of air pollution risk in shaping pro-environmental orientations. However, these effects appear to be more pronounced among urban residents, suggesting that limited research has examined how air pollution risk is perceived and responded to in ecologically significant tourist destinations like Jeju. Furthermore, research efforts to understand the perspectives of outdoor leisure participants on environmental policies are still lacking (Gifford, 2011; Mu et al., 2022), which makes the research hypotheses of this study meaningful and timely.

Despite the growing public concern about air quality in Korea, few studies have integrated risk perception of fine dust with psychological theories of restorative environments or explored how these factors jointly influence support for environmental policies. Environmental policy plays a crucial role in facilitating and sustaining pro-environmental behaviors. Policies aimed at mitigating pollution and preserving natural spaces can provide individuals with more opportunities to engage in sustainable practices (Thøgersen and Crompton, 2009). Furthermore, public support for environmental policies is essential for their effective implementation and long-term success (Sharpe et al., 2021). Studies in environmental psychology suggest that people's level of environmental concern is closely related to their connection to nature and experiences in restorative environments (Marchetti et al., 2024; Guazzini et al., 2025). Individuals who experience cognitive restoration in natural settings may develop stronger motivations to support environmental policies that aim to protect such spaces (Hartig et al., 2001).

This study addresses a significant research gap by situating the investigation within Jeju Gotjawal Provincial Park—a unique ecological landscape under increasing tourism pressure—offering context-specific insights that can inform both environmental management and public health strategy.

**Figure 1:**
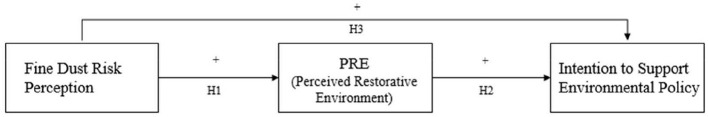
Hypothesized model.

Therefore, this study contributes to the literature by integrating fine dust risk perception, Perceived Restorative Environment (PRE), and environmental policy support intention within a single structural framework. By focusing on visitors to Jeju Gotjawal Provincial Park—a rare, ecologically preserved site in a region frequently affected by air pollution—this research provides novel insight into how environmental risk and restorative experience interact in shaping environmental attitudes and behaviors. Based on these findings, this study examines the relationship between fine dust risk perception, PRE, and intention to support environmental policies among visitors at Jeju Gotjawal Provincial Park. Additionally, to provide a more comprehensive understanding of the psychological mechanisms involved, the study examines whether PRE mediates the relationship between fine dust risk perception and policy support. Specifically, the research hypotheses and the research model (Figure 1) proposed in this study are presented as follows:

*Hypothesis 1: As park visitors' fine dust risk perception increases, their perceived restorative environment (PRE) will increase*.

*Hypothesis 2: As park visitors' PRE increases, their intention to support environmental policies will increase*.

*Hypothesis 3: As park visitors' fine dust risk perception increases, their intention to support environmental policies will increase*.

*Hypothesis 4: The relationship between fine dust risk perception and intention to support environmental policies will be mediated by PRE*.

## 2 Literature review

### 2.1 Fine dust risk perception

Fine dust, or particulate matter (PM2.5), is a significant environmental and public health concern, particularly in South Korea, where transboundary pollution and industrial emissions contribute to its prevalence (Vohra et al., 2021). Risk perception refers to how individuals evaluate the severity and potential harm of environmental threats such as fine dust pollution. Studies suggest that higher risk perception of fine dust is associated with increased concern for health and environmental well-being (Kim et al., 2020). Individuals who perceive fine dust as a severe risk tend to adjust their behaviors, such as limiting outdoor activities or seeking cleaner environments (Kim and Shin, 2021). By evaluating that outdoor activities rather deteriorate health, they choose to stop outdoor physical activities (Kim and Kim, 2020). This reflects fine dust risk perception, a type of environmental risk perception based on sensory information (Lee and Kim, 2017). Health-related behaviors, such as ceasing outdoor activity, are examples of responses that may contribute to disease and stress (Park and Han, 2021). Moreover, risk perception has been linked to greater environmental awareness and a willingness to engage in protective behaviors, including policy support and advocacy for air quality improvements (Lee and Kim, 2017).

### 2.2 Perceived restorative environment

Natural environments, particularly green spaces and forests have been widely recognized for their restorative qualities, which contribute to psychological well-being and stress reduction (Kaplan and Kaplan, 1989; Byrka et al., 2010). The concept of the PRE refers to an individual's perception of nature's ability to restore cognitive and emotional balance (Hartig et al., 1997). Research suggests that exposure to natural environments fosters relaxation, enhances mood, and improves cognitive function (Marselle et al., 2013). A substantial body of research highlights that exposure to natural environments provides significant physical and mental health benefits for urban residents, including stress reduction, attention restoration, positive emotional states, and disease prevention (Hunter et al., 2019; Kajosaari and Pasanen, 2021). Furthermore, air pollution concerns, such as fine dust, can drive individuals to seek restorative natural settings to counteract the psychological distress associated with pollution (Bratman et al., 2019). Studies have shown that individuals with higher fine dust risk perception are more likely to value nature's restorative benefits, reinforcing the role of green spaces as essential for mental and physical well-being (Seok et al., 2024).

### 2.3 Intention to support environmental policies

Public support for environmental policies is critical for implementing sustainable measures to mitigate air pollution and preserve natural ecosystems (Sharpe et al., 2021). The intention to support environmental policies is influenced by several factors, including personal experiences with environmental issues, risk perception, and interactions with restorative environments (Marchetti et al., 2024). Individuals who perceive natural environments as restorative are more likely to develop pro-environmental attitudes and behaviors (Schuttler et al., 2018). Research indicates that positive experiences in nature not only enhance well-being but also foster greater environmental consciousness, leading to increased support for policies aimed at conservation and pollution reduction (Dean et al., 2019). Therefore, emotion from positive experiences is a reliable motivator for pro-environmental behavior (Roeser, 2012). Corral-Verdugo (2013) even found that emotions have a higher impact on pro-environmental behavior than intentions. Moreover, the relationship between fine dust risk perception and policy support is often mediated by the restorative experience of nature, as those who seek refuge in natural settings develop a stronger commitment to sustainability and environmental advocacy (Byrka et al., 2010; Guazzini et al., 2025).

In summary, fine dust risk perception influences individuals' inclination to seek restorative environments, which in turn fosters stronger support for environmental policies. Understanding these relationships can provide valuable insights for policymakers and practitioners to design strategies that promote public well-being and environmental sustainability.

## 3 Methods

### 3.1 Data collection

Data collection was conducted at Jeju Gotjawal Provincial Park in October 2021. South Korea experiences four distinct seasons, and Jeju Island's average temperature in October is 15.5 °C (59.9°F), making it one of the most pleasant months for outdoor activities. October was chosen as the data collection period because it marks the peak visitor season for the park, coinciding with the ideal autumn weather conditions. Although official visitor statistics were not available from Jeju Gotjawal Provincial Park, the park management office recommended October as the month with the highest number of visitors, estimating approximately 25,000 visitors in October 2021. Furthermore, Jeju Island recorded 1.2 million tourists in October 2021, the highest number of visitors during the year (Jeju Tourism Association, 2021). Gotjawal forests are unique ecosystems formed on irregular basaltic lava terrain from past volcanic eruptions. These forests are home to diverse and endemic flora and fauna found only in Jeju. Gotjawal Provincial Park is one of the four major Gotjawal regions, located in Daejeong-eup, Seogwipo-si, covering an area of approximately 1.54 million square meters.

For this study, purposive sampling was employed to select participants who were visiting the park for hiking. The park offers five hiking courses, and data collection was conducted at the only entrance/exit point of the hiking trails. Four trained research assistants were stationed at this location to approach visitors, explain the study's purpose, and obtain their consent before distributing the survey. All surveys were conducted at a desk located just inside the park entrance/exit, targeting visitors who had completed their park visit to ensure that responses reflected actual restorative experiences. Participants were provided with self-administered questionnaires, which they completed on-site. Data collection took place between 9:00 a.m. and 2:00 p.m., corresponding to the park's opening hours. The peak hiking period, as advised by park managers, was between 10:00 a.m. and 1:00 p.m. A total of 408 complete questionnaires were collected and used in the final analysis.

Although this study used purposive sampling, the method was well-suited to the study aims and setting. Jeju Gotjawal Provincial Park attracts a specific group of visitors who seek nature-based experiences and are likely to be concerned about environmental issues such as air quality. Targeting this population allowed us to examine how fine dust risk perception and perceived restorativeness relate to environmental policy support in a meaningful real-world context. In leisure and environmental psychology research, purposive sampling is frequently used when the goal is analytical rather than statistical generalization, especially when studying psychologically relevant subpopulations (Patton, 2015; Veal, 2017). Thus, the approach was consistent with established research standards in similar fields.

Despite the data being collected in the second half of 2021, recent studies and reports indicate that the characteristics of visitors to Jeju have remained relatively stable over the past 4 years. For instance, the number of visitors to Gotjawal Provincial Park has consistently remained around 200,000 annually since 2020 (Kim et al., 2022). A government-sponsored survey also found that key attributes of Jeju visitors—such as travel patterns, spending, and revisit rates—have shown minimal fluctuation in recent years (Jeju Tourism Organization, 2023). Moreover, ecological tourism evaluations conducted in 2023 reported satisfaction scores above 9 out of 10 among Gotjawal visitors, reflecting consistently high evaluations of the park experience (Jeju Tourism Organization, 2023). Additionally, interviews with park officials confirmed that trail conditions and management practices have not changed significantly over the past 5 years. These findings collectively support the ongoing validity of our 2021 dataset despite the time lag in publication.

### 3.2 Measures

All items used in the study are presented in Table 1. First, fine dust risk perception consisted of five items based on prior research to measure the degree of individual risk perception for fine dust (Kim and Shin, 2021; Lee and Kim, 2017). The Fine Dust Risk Perception scale was validated for reliability and validity in the studies by Choi (2023) and Park and Kim (2019), and it consists of items that are appropriate for measuring the perception of fine dust risk among Koreans. Second, PRE was measured by four survey items drawn from the PRS (Hartig et al., 1997). The PRE scale used in this study was adapted from Hartig et al. (2014), which originally included 16 items across four subdimensions: Being Away, Fascination, Coherence, and Compatibility. However, previous research in Korea demonstrated that a shortened five-item version is valid and reliable in similar contexts (Kang and Seo, 2020). In this study, one item was excluded due to low factor loading, resulting in a final set of four items. These items captured the positive psychological feelings of restoration perceived from the park (e.g., “Jeju Gotjawal Provincial Park is a place where I can get out of my daily life and relax and think about my favorite things”). Lastly, the intention to support environmental policy was measured using items adapted from the literature (Leiserowitz, 2006) and included dimensions of environmental policy support (three survey items). The policy support scale used in this study was adopted from previous studies that validated its reliability and validity in measuring Koreans' intention to support policies aimed at addressing environmental issues (Oh and Yun, 2022). Additionally, the survey included questions about respondents' sociodemographic information such as age, gender, income, and education level.

**Table 1:** Means, standard deviations, internal consistency, and factor loadings for fine dust risk perception, perceived restorative environment, and intention to support environmental policy.

**Variables/survey items**	***SNF***				***a***
	**M**	**SD**	λ	**SE**	
**Fine dust risk perception**					
a. Fine dust causes diseases such as respiratory illnesses, lung disease, and cancer.	4.57	0.60	0.82	0.02	0.829
b. Fine dust is highly harmful to both humans and nature.	4.56	0.61	0.94	0.02	0.940
c. Fine dust has lasting effects on future generations.	4.63	0.60	0.83	0.02	0.834
d. Fine dust causes economic damage, including impacts on crops, vehicle filters, and air purifiers.	4.55	0.60	0.88	0.02	0.885
e. Fine dust leads to communication disruptions, such as interference with air travel operations.	4.19	0.87	0.64	0.04	0.635
**Perceived restorative environment**	
a. The park is a large and attractive place where one can explore nature and satisfy their curiosity.	4.45	0.68	0.62	0.03	0.615
b. The park is well-organized, free from congestion, and not disorderly.	4.25	0.78	0.75	0.03	0.756
c. The park offers a spacious and open environment with no restrictions on movement.	4.05	0.94	0.80	0.04	0.809
d. In the park, it is easy to navigate, move around, and engage in activities of personal interest.	4.13	0.81	0.82	0.03	0.822
**Intention to support environmental policy**	
a. I support the government's implementation of fine dust countermeasures.	4.30	0.79	0.81	0.03	0.819
b. I believe it is desirable to expand the application of the government's environmental policies.	4.27	0.85	0.88	0.03	0.880
c. I prefer that the benefits of the government's fine dust and environmental policies be provided to me and my immediate surroundings first.	4.18	0.81	0.72	0.03	0.723

Scales ranged from 1 (strongly disagree) to 5 (strongly agree).

Fit statistics: χ^2^ = 178.905, df = 51, RMSEA = 0.076, NNFI = 0.964, CFI = 0.972.

To minimize measurement bias, the survey utilized validated scales widely employed in environmental psychology and risk perception research (e.g., Hartig et al., 1997; Kim and Shin, 2021; Leiserowitz, 2006). Internal consistency was confirmed via Cronbach's alpha, and CFA results showed satisfactory model fit, supporting construct validity. To reduce social desirability bias, all responses were collected anonymously, and participants were assured of the confidentiality and voluntary nature of the study (Podsakoff et al., 2003). The survey items focused on participants' immediate perceptions and affective evaluations of the park, reducing the likelihood of recall bias typically associated with retrospective self-reports (Bradburn et al., 1987). Although the scales used were concise, they were adapted from validated instruments and demonstrated acceptable reliability and construct validity in CFA, ensuring their appropriateness for field-based data collection.

### 3.3 Analyses

We used confirmatory factor analysis (CFA) to validate the theorized factor structure of our measurement model, and the goodness-of-fit index was used to confirm the variable composition. The measurement model was assessed using the following goodness-of-fit indices: root mean square error of approximation (RMSEA under.10) (MacCallum et al., 1996), non-normed fit indices (NNFI greater than 0.90) (Hu and Bentler, 1998), and comparative fit indices (CFI greater than 0.95) (Hu and Bentler, 1998). All analyses were conducted using LISREL 8.70. In addition, a bootstrap analysis was conducted to verify the mediating effect. This analysis was performed using the PROCESS macro in SPSS 30.0. We set bootstrap confidence interval (CI) at 95% and number of bootstrap samples was 5,000. If zero was not included in the interval of 95% CI, it indicated that the mediating effect was significant (MacKinnon, 2008).

## 4 Results

### 4.1 Correlation between variables

Table 2 shows the results of the Pearson correlation analysis. There were all positive correlations between fine dust risk perception, PRE, and intention to support environmental policy. In addition, the correlation coefficient between each variable was between 0.326 and 0.428 (*p* < 0.01); there was no problem with the construct validity (Kline, 2013).

**Table 2:** The results of correlation analysis.

**Variables**	**1**	**2**	**3**
Fine dust risk perception (1)	1		
Perceived restorative environment (2)	0.326^**^	1	
Intention to support environmental policy (3)	0.428^**^	0.363^**^	1

^**^p < 0.01.

### 4.2 Descriptive analysis

As shown in Table 3, a total of 62.00% of respondents were women. The gender distribution in the sample, with a majority of female respondents (62%), reflects patterns commonly observed in nature-based recreation participation (Jeju National University, 2020; Gobster, 2005). The respondents' age ranged from 10 to 79, and the mean age was 46.72 (SD = 13.84). About half of the respondents were university graduates (50.50%). About 20.50% of respondents' annual average income was more than 70,000,000 KRW (approximately USD 65,800). The mean and standard deviation of all items of the variables included in the research model are presented in Table 3.

**Table 3:** Socio-demographic characteristics.

**Characteristics**	**Category**	**Valid percent**
Gender	Male	38.00
	Female	62.00
Age	Mean = 46.72 (SD = 13.84)	
Education	Elementary—high school	19.10
	Two-year college	10.10
	University	50.50
	Master's degree and more	20.30
Annual income	< KRW 10,000,000 (approx. USD 9,400)	9.60
	KRW 10,000,000–19,990,000	4.50
	KRW 20,000,000–29,990,000	12.40
	KRW 30,000,000–39,990,000	16.40
	KRW 40,000,000–49,990,000	15.90
	KRW 50,000,000–59,990,000	12.40
	KRW 60,000,000–69,990,000	8.30
	>KRW 70,000,000	20.50
Total		*n* = 408

### 4.3 Gender differences in key variables

To examine whether gender influenced the fine dust risk perception, PRE, and intention to support environmental policy, independent samples *t*-tests were conducted. The results indicated no significant gender differences in PRE (*t* = −1.873, *p* =0.062), fine dust risk perception (*t* = −1.502, *p* =0.134), or intention to support environmental policy (*t* = −0.543, *p* =0.587). Although the sample included a slightly higher proportion of female respondents (approximately 62%), independent samples *t*-tests revealed no significant gender differences across the three main study variables. This suggests that the gender imbalance in the sample does not pose a substantial threat to the validity of the findings.

### 4.4 Model testing for pooled sample

**Figure 2:**
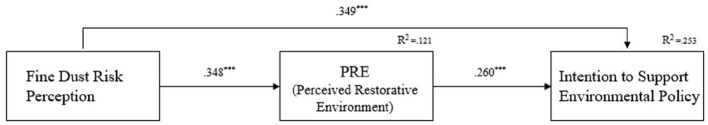
Final model estimation.

For the pooled sample (n = 408), both the measurement model (χ^2^ = 248.775, df = 52, RMSEA =0.079, NNFI =0.956, CFI =0.958) and structural model (χ^2^ = 114.946, df = 49, RMSEA =0.057, NNFI =0.981, CFI =0.986) showed an acceptable fit for the data (see Table 4). All fit indices satisfied the recommended thresholds, indicating that the models were appropriate representations of the data. The internal consistency of the factors was confirmed with Cronbach's alpha values exceeding 0.60 for all variables (Hair et al., 1998) (see Table 1). All three hypotheses were supported (see Table 5). Figure 2 presents a summary of the research model. First, the fine dust risk perception of visitors at Jeju Gotjawal Provincial Park positively influenced PRE (H1; β = 0.348, *p* < 0.001) and 12.10% of the variance in PRE was explained by fine dust risk perception. Second, the PRE positively influenced the intention to support environmental policy (H2; β = 0.260, *p* < 0.001). Third, the fine dust risk perception had a positive effect on intention to support environmental policy (H3; β = 0.349, *p* < 0.001). The variance in on intention to support environmental policy was accounted for by fine dust risk perception and PRE by 25.30% Finally, we concluded that there was a structural relationship among fine dust risk perception, PRE, and intention to support environmental policy among the Jeju Gotjawal Provincial Park visitors.

**Table 4:** Fit indices for the baseline models.

**Model**		**χ^2^**	**df**	**RMSEA**	**NNFI**	**CFI**
Pooled (*n* = 408)	Measurement model	248.775	52	0.079	0.956	0.958
Pooled (*n* = 408)	Structural model	114.946	49	0.057	0.981	0.986

**Table 5:** Hypotheses testing (*n* = 408).

**Hypothesis**	**Predictor**	**Path**	**D.V**.	***R* ^2^**	**β**	**S. E**
H1	Fine dust risk perception	→	PRE	0.121	0.348^***^	0.059
H2	PRE	→	Intention to support environmental policy	0.253	0.260^***^	0.063
H3	Fine dust risk perception	→	Intention to support environmental policy		0.349^***^	0.064

^***^p < 0.001.

We further analyzed the indirect effects to examine whether PRE was a significant mediator of the relationship between the fine dust risk perception and intention to support environmental policy. Bootstrapping results provided support for the causal relationship among the variables (see Table 6). None of the confidence intervals for the indirect effects included zero, indicating that the indirect effects in the hypothesized model were statistically significant. These analyses empirically demonstrated that fine dust risk perception had a significant positive indirect effect on the intention to support environmental policy through PRE [indirect effect = 0.0619, 95% CI (0.0358, 0.0923), *p* < 0.001]. Therefore, H4 was supported, indicating that PRE significantly mediates the relationship between fine dust risk perception and intention to support environmental policy.

**Table 6:** Indirect effect of PRE on the relationship between fine dust risk Perception and Intention to Support Environmental Policy using bootstrapped 95% confidence intervals (lower and upper bounds).

**Hypothesis**	**Path**	**Indirect effect**	**Direct effect**	**S. E**	**95% C.I**.
H4	Fine dust risk perception → PRE → intention to support environmental policy	0.0619^***^	0.2635	0.0145	(0.0358, 0.0923)

^***^p < 0.001.

## 5 Discussion

This study examined the relationship between fine dust risk perception, PRE, and intention to support environmental policy among visitors to Jeju Gotjawal Provincial Park. The findings provide several theoretical and practical insights into how environmental concerns influence individuals' experiences in natural settings and their subsequent environmental attitudes and behaviors. This study offers a unique contribution by integrating fine dust risk perception, perceived restorativeness, and environmental policy support into a single structural model—an approach that has received limited attention in previous research. While prior studies have explored these variables separately, our model clarifies how psychological restoration from nature not only mediates environmental concern but also motivates policy support. This research adds a novel insight into the psychological mechanisms linking environmental risk to civic engagement in the Korean context, where air pollution remains a prominent issue.

### 5.1 Theoretical implications

First, the results support Hypothesis 1, indicating that as visitors' fine dust risk perception increases, their PRE also increases. This suggests that individuals who perceive fine dust as a serious environmental and health risk are more likely to appreciate and seek natural environments like Gotjawal forests as restorative spaces. This aligns with previous research emphasizing the role of environmental threats in shaping human-nature interactions (Bratman et al., 2019; Hartig et al., 2014). When urban air pollution levels rise, people increasingly recognize the benefits of clean and green environments, reinforcing the psychological significance of natural areas as places for stress relief and well-being (Seok et al., 2024).

Furthermore, the findings confirm Hypothesis 2, showing that a perception of restorativeness in natural environments positively influences individuals' support for environmental policies. This aligns with the restorative environment theory, which suggests that individuals who experience psychological and cognitive benefits from nature develop stronger pro-environmental attitudes and behaviors (Kaplan and Kaplan, 1989; Marselle et al., 2013). This finding suggests that people's nature-based experiences are not isolated leisure episodes, but significant psychological events that can shape their attitudes toward social and environmental change.

Additionally, the findings confirm Hypothesis 3, showing that a perception of fine dust risk positively influences intention to support environmental policy. This aligns with the previous research emphasizing the role of environmental risk perception. Specifically, risk perception has been linked to greater environmental awareness and willingness to engage in protective behaviors, including policy support and advocacy for air quality improvements (Lee and Kim, 2017).

Beyond these direct effects, the study also identified significant indirect effects through perception of restorativeness in natural environments the relationship between fine dust risk perception and intention to support environmental policy. The finding aligns with Schuttler et al. (2018), who emphasized that individuals' affective and experiential connection to nature plays a crucial role in fostering environmental concern and pro-environmental behaviors. Their study demonstrated that positive personal experiences in nature, such as feelings of restoration and emotional connectedness, can enhance individuals' pro-environmental behavior. In line with this, the present findings suggest that when individuals perceive nature as psychologically restorative, this experience strengthens the influence of environmental risk perception on policy support. In this sense, perception of restorativeness serves as an emotional and cognitive gateway that transforms abstract environmental threats into concrete behavioral intentions.

Additionally, this model is particularly meaningful in the South Korean context, where chronic air quality concerns have shaped urban residents' relationship with nature. By incorporating risk perception, psychological response, and policy support in one framework, this study aligns with broader theoretical models of pro-environmental behavior that integrate affective, cognitive, and behavioral domains (Stern, 2000).

### 5.2 Practical implications

These findings offer important applications for environmental policymakers, urban planners, and tourism management. First, given the increasing concerns about air pollution, authorities should promote nature-based solutions to enhance public well-being. By increasing access to clean, natural environments, such as protected parks and urban green spaces, policymakers can help mitigate the negative effects of fine dust exposure while also fostering greater environmental engagement.

Second, environmental policies should incorporate public education programs that emphasize the link between nature experiences and sustainability. Raising awareness about the psychological and physical benefits of natural spaces could strengthen public support for conservation policies and encourage responsible behavior. For example, initiatives that highlight the health benefits of forests, clean air, and biodiversity conservation could enhance individuals' commitment to environmentally friendly practices and policies (Sharpe et al., 2021). In addition, health-focused campaigns could be integrated with park promotion initiatives to explicitly link environmental well-being with public health outcomes. Targeted interventions for populations most vulnerable to fine dust, such as older adults, children, or those with respiratory conditions, could further strengthen both health benefits and support for environmental policies.

Third, nature-based tourism programs—particularly in ecologically vulnerable destinations such as Jeju—can be designed to include environmental messaging and stewardship elements. By emphasizing the restorative value of nature through interpretive signage, guided experiences, or visitor feedback campaigns, park managers can enhance both visitor well-being and policy consciousness.

Moreover, for urban populations experiencing eco-anxiety or pollution fatigue, structured nature exposure can be introduced not merely as recreation but as a form of mental health intervention and behavioral activation. These programs could be coordinated with public health institutions, contributing to integrated environmental-health policy.

### 5.3 Limitations and future research

While this study provides valuable insights, several limitations should be acknowledged. First, the data were collected from a single site (Jeju Gotjawal Provincial Park), limiting the generalizability of the findings to other populations and geographical contexts. This study was limited to a single site, Jeju Gotjawal Provincial Park, with purposive sampling, which restricts the generalizability of the findings. The unique ecological setting and cultural context of fine dust concerns in Korea may have shaped participants' responses. Future studies should replicate this work across different cultural and environmental contexts to clarify whether these relationships are universal or context-specific. Future studies could expand this research to include multiple natural settings across South Korea to validate the results in diverse environmental contexts. The unique ecological and cultural context of Korea may have shaped participants' responses, so future studies should test these relationships in diverse settings to assess their generalizability.

Second, the sample consisted of a higher proportion of women (62%). Although no significant gender differences were observed, this imbalance may have influenced the way participants experienced restorativeness and supported environmental policies. Previous research suggests that women are more likely to experience eco-anxiety (Goudet et al., 2023), a newly emerging mental health concern in the context of climate change, which may partly shape their support for environmental policies. Future research should replicate these findings with more gender-balanced samples to enhance the generalizability of the results.

Third, this study relied on self-reported survey data, which may be subject to social desirability and recall biases. Although validated measurement scales were used and anonymity was assured to reduce response bias, the potential for subjective bias remains. To enhance the robustness of findings, future research could integrate objective indicators such as real-time air quality data (e.g., PM2.5 levels) or physiological markers (e.g., heart rate variability), which would allow for multimethod triangulation and stronger validation of the observed relationships (Hunter et al., 2019).

Fourth, while the structural model revealed significant associations among fine dust risk perception, perceived restorativeness, and environmental policy support, the cross-sectional nature of the data prevents definitive causal claims, and the observed mediation should be understood as associative rather than causal. Therefore, the results should be understood as structural relationships rather than definitive causal pathways. Future longitudinal or experimental research is recommended to validate these mediation effects over time.

Fifth, another limitation concerns the use of the reduced four-item PRE scale. While this shorter version has demonstrated acceptable reliability and practical utility in the Korean context, it may not fully capture the multidimensional nature of the original PRS (i.e., Being Away, Fascination, Coherence, and Compatibility). Thus, certain aspects of restorative experience might have been underrepresented. Future research should consider employing the full or intermediate versions of the PRS to ensure broader construct coverage and to compare results across different measurement approaches.

Lastly, while this study focused on fine dust risk perception, future research could explore other environmental stressors (e.g., climate change anxiety, urban noise pollution) and their impact on restorative experiences and environmental policy engagement. Understanding how multiple environmental concerns interact to influence behavior could provide a more comprehensive framework for promoting sustainability. In addition, future studies could examine how individual characteristics—such as gender, age, or socioeconomic status—moderate these relationships. Exploring demographic differences may yield important insights into the diverse ways people perceive environmental risks and engage in policy support. Although no significant gender differences were found in the present study, it remains important to consider the gender composition of the sample when interpreting the findings, as it may affect the generalizability of results. Nonetheless, the absence of gender effects suggests that the findings are robust across gender groups, enhancing their applicability to broader populations.

## 6 Conclusion

This study findings indicate that fine dust risk perception is positively associated with perceived restorativeness in nature, which in turn enhances individuals' support for environmental policies. These findings highlight the importance of nature exposure in shaping pro-environmental attitudes and underscore the need for stronger environmental policies and conservation efforts. As societies increasingly face environmental crises, understanding how individuals' emotional experiences in nature influence their environmental attitudes becomes essential. Engaging the public through nature may be a vital step toward collective climate resilience, sustainable behavior, and transformative policy change.

## Data Availability

The original contributions presented in the study are included in the article/supplementary material, further inquiries can be directed to the corresponding author.
